# Isotropic fluid structures and role of non-metricity on their dynamics governed by an electric charge: A theoretical study

**DOI:** 10.1371/journal.pone.0343123

**Published:** 2026-03-20

**Authors:** Tayyab Naseer, M. Sharif, M. Waqas, J. Andrade, Ahmed Emara

**Affiliations:** 1 Department of Mathematics and Statistics, The University of Lahore, Lahore, Pakistan; 2 Research Center of Astrophysics and Cosmology, Khazar University, Baku, Azerbaijan; 3 Escuela Superior Politécnica de Chimborazo, (ESPOCH), Riobamba, Ecuador; 4 Department of Electrical Engineering, University of Business and Technology, Jeddah, Saudi Arabia; 5 Department of Engineering Mathematics, and Physics, Faculty of Engineering, Alexandria University, Alexandria, Egypt; University of Sharjah, UNITED ARAB EMIRATES

## Abstract

This research explores the dynamics of compact stellar objects characterized by the Kohler-Chao-Tikekar and Tolman IV spacetimes under the effects of an electric charge within the framework of f(Q) gravity, where *Q* indicates the non-metricity. We start our analysis with the assumption of a static spherical line element and formulate the field equations corresponding to an isotropic interior fluid setup. We then construct two novel solutions under the two above-mentioned metrics which involve multiple unknown constants. These constants are calculated using junction conditions at the boundary, where we match our interior solutions with the exterior Reissner-Nordström line element. Afterwards, we analyze specific conditions that must be met to ensure the feasibility of compact models in real-world applications. The graphical analysis is performed under a particular star candidate, namely LMC X-4 with several choices of the considered model parameters. Under certain chosen values, our results show that both stellar solutions are in good agreement with the physically existence criterion. In light of the current findings, we deduce that our work significantly advances the understanding of how the interior fluid dynamics of compact stars are influenced by the symmetric teleparallel gravity, offering vital information for further research in astrophysics.

## 1 Introduction

The early twentieth century witnessed a fundamental transformation in gravitational physics when Albert Einstein introduced his general theory of relativity (GR) in 1915. This ground-breaking framework redefined our conception of spacetime geometry and its dynamic interaction with matter and energy. Contemporary cosmological research has established that the universe undergoes accelerated expansion, an observational result requiring the existence of dark energy, an enigmatic cosmological constituent that currently lacks complete theoretical understanding [1,2]. While the cosmological constant (*λ*) successfully models cosmic expansion [3], it encounters significant challenges – notably the coincidence problem [4] and fine-tuning discrepancy [5] in GR. Consequently, GR may not represent the optimal framework for gravitational interactions at cosmological scales. Given our incomplete understanding of cosmic acceleration, investigating alternative gravity theories remains essential. These studies offer promising alternatives to GR and could potentially address several of its current limitations. Recent decades have seen vigorous debate around modified gravity theories, as researchers employ them to elucidate the large-scale structure and evolution of the universe [6–9]. Consequently, a significant body of work has emerged that calculates the maximum mass and compactness of neutron stars in various modified gravity models, testing these predictions directly against the constraints of GR [10–12].

When the connection’s antisymmetric component (torsion *T*) is non-vanishing, both teleparallel equivalent GR and its f(T) extensions diverge from GR while preserving metric compatibility. This framework consequently suggests a third gravitational formulation, the symmetric teleparallel equivalent GR (STEGR) – where the metricity condition is abandoned in favor of a novel geometric property called non-metricity. Consequently, the STEGR action is formulated exclusively via non-metricity, yet it exactly reproduces GR dynamics. For a comprehensive examination of these three unique geometric formulations of gravity, the authors characterize them as gravity’s “geometrical trinity” in reference [13]. This theory admits natural extensions through general non-metricity dependent functions in the action, analogous to the f(R) and f(T) formulations in modified gravity theories. This generalization leads to f(Q) gravity, a framework that has attracted significant research attention in recent literature [14–36]. This investigation examines the minimal f(Q) functional formulation and its implications in the context of compact stellar structures.

General theory of relativity employs Riemannian geometry to model spacetime’s structure and the Levi-Civita connection to describe dynamics within it [37]. The Levi-Civita connection is predicated on the assumption of pure curvature, requiring the vanishing of both torsion and non-metricity. The choice of connection on the manifold whether Levi-Civita or otherwise dictates the resulting theory of gravity, producing formulations that differ in their details but may be conceptually related. The inclusion of non-zero torsion and non-metricity provides a foundation for constructing a broad spectrum of gravitational theories beyond GR. One can derive the teleparallel counterpart to GR by imposing the conditions of zero curvature and zero non-metricity, but relaxing the constraint on torsion [38–40]. Conversely, STEGR, the geometric foundation of GR [19,41,42], and its extension to f(Q) gravity, are formulated in a flat, torsion-less spacetime where gravity is attributed entirely to non-metricity. In this framework, the gravitational interaction is characterized entirely by the non-metricity scalar [43]. The primary motivation for studying f(Q) gravity is its potential to address key cosmological issues, particularly in explaining the observed late-time cosmic acceleration without invoking dark energy [44–46]. By promoting the action to a general function of the non-metricity scalar, this theory generalizes GR and provides a broader framework for exploring gravitational effects.

To determine how matter couples to this theory, Harko et al. [47] analyzed it through the lens of both power-law and exponential models. Lazkoz et al. [48] conducted an observational analysis of various f(Q) models using redshift parametrization. Their work assessed the viability of these models as alternatives to *λ*-CDM in explaining late-time cosmic acceleration. Mandal et al. [49] employed energy conditions to analyze this theory of gravity, seeking models that remain consistent with the observed rapid cosmic expansion. Lin and Zhai [30] examined the effects of f(Q) gravity on compact stars, finding that a positive model parameter decreases stellar mass, whereas a negative value increases it. Mandal and Sahoo [50] computed the equation of state and Hubble parameter, demonstrating that the f(Q) model’s dynamics diverge significantly from those of the CDM model. Lymperis [51] studied the theory’s cosmic implications through its effective dark energy sector, while Koussour et al. [52] analyzed the evolution of key cosmological parameters within the same framework. Araujo and Fortes [53] applied these gravitational models to polytropic stars to study the impact of non-metricity on stellar dynamics. Within this gravitational framework, Sharif and Ajmal developed interacting models of generalized ghost pilgrim and generalized ghost dark energy [54], in addition to examining non-interacting scenarios with pilgrim dark energy and its generalized ghost variant [55].

The analysis of stellar structures and gravitational collapse has been a central pursuit in astrophysics now a days. The gravitational collapse of a star culminates in a compact remnant – a white dwarf, neutron star, or a black hole – depending primarily on its initial mass. The comprehensive theoretical and observational examination of these compact remnants has established their study, especially of neutron stars, as a particularly intricate and dynamic area of contemporary astrophysical research. The study of these compact objects leverages a powerful synergy of theory (e.g., hydrodynamic simulations, quantum field approximations) and observation (e.g., X-ray satellites, gravitational wave detectors, multi-wavelength telescopes), providing deep insights into the physics of matter under the most extreme conditions. The investigation of neutron stars stands as one of modern astrophysics’ most multifaceted and captivating pursuits. It encompasses phenomena like pulsar emissions, superfluid interiors, and potential quark matter phases, all arising from the catastrophic implosion of massive stellar cores that have exhausted their nuclear fuel and can no longer support themselves against gravitational collapse.

Exact analytical solutions to the gravitational field equations remain exceptionally challenging, especially those satisfying all physical viability criteria for being realistic stellar configurations. Delgaty and Lake [56] systematically evaluated a large number of distinct solutions, determining that merely a few of them satisfied all fundamental physical requirements for realistic stellar models. The study of relativistic stellar interiors through the field equations’ exact solutions continue to be a vital research frontier. Rahman and Visser [57] established a complete classification of all static, spherically symmetric perfect-fluid space-time metrics. These findings offer particularly significant insights for resolving fundamental uncertainties in the dense matter equation of state within compact relativistic objects. Martin and Visser [58] significantly extended the Buchdahl stability limit through rigorous constraints on multiple physical parameters characterizing static perfect-fluid configurations. The derived constraints incorporated four fundamental aspects: *(i)* an interior spacetime geometry via metric component bounds, *(ii)* proper acceleration field characteristics, *(iii)* correlated radial density-pressure relationships, and *(iv)* the compactness function 2m(r)/r throughout the stellar interior. These generalized constraints establish a rigorous theoretical foundation for examining relativistic stellar equilibrium, especially when addressing uncertainties in ultra-dense matter physics. These bounds offer critical diagnostics for assessing equation of state viability in extreme gravity regimes. Building on this framework, Lake [59] introduced a novel algorithm that generates all regular static spherically symmetric perfect-fluid solutions through a single monotone function satisfying boundary conditions. This method guarantees both solutions’ regularity and physical viability while enabling the systematic derivation of several new exact solutions. A subsequent advancement reformulated the approach using physically transparent variables [60]. This reformulation not only reveals fundamental connections between known solutions but also facilitates the construction of new physically admissible static solutions to Einstein’s field equations.

This work investigates the modified field equations in f(Q) gravity through the Kohler-Chao-Tikekar and Tolman IV spacetime geometries, analyzing their implications for compact object structures. The paper is organized as follows: Section 2 presents the theoretical foundations of f(Q) gravity, including its modified field equations which are derived for spherically symmetric geometries using both the above-mentioned ansatz. Section 3 examines compact stars’ structure, with particular emphasis on junction conditions at the stellar surface. Sections 4 and 5 examine the physical properties of the derived stellar models, including a detailed stability analysis. The concluding remarks and implications of our results are presented in Section 6.

## 2 Structure of f(Q) gravity and spherical geometry

The f(Q) theory of gravity uses non-metricity as its fundamental geometric property [61]


$$Q_{\tau \eta \chi }=-g_{\eta \chi ,\tau }+g_{\chi \xi }{\hat{\Gamma }}_{\eta \tau }^{\xi }+g_{\xi \eta }{\hat{\Gamma }}_{\chi \tau }^{\xi }.$$ (1)

The metric tensor in this framework is expressed as $g_{\eta \chi }$, while the affine connection appears as ${\hat{\Gamma }}_{\eta \chi }^{\tau }$ which decomposes into three unique parts [62]


$${\hat{\Gamma }}_{\eta \chi }^{\tau }=\Gamma_{\eta \chi }^{\tau }+{\mathbb{C}}_{\;\eta \chi }^{\tau }+{𝕃}_{\;\eta \chi }^{\tau }.$$ (2)

The Levi-Civita connection in this context is expressed as


$$\Gamma_{\eta \chi }^{\tau }=\frac{1}{2}g^{\tau \xi }(g_{\xi \chi ,\eta }+g_{\xi \eta ,\chi }-g_{\eta \chi ,\xi }),$$ (3)

The contortion tensor is mathematically represented as


$${\mathbb{C}}_{\;\eta \chi }^{\tau }={\hat{\Gamma }}_{\left[\eta \chi \right]}^{\tau }+g^{\tau \xi }g_{\eta \kappa }{\hat{\Gamma }}_{\left[\chi \xi \right]}^{\kappa }+g^{\tau \xi }g_{\chi \kappa }{\hat{\Gamma }}_{\left[\eta \xi \right]}^{\kappa }.$$ (4)

In contrast to contortion, the disformation tensor emerges as


$${𝕃}_{\;\eta \chi }^{\tau }=\frac{1}{2}g^{\tau \xi }(Q_{\chi \eta \xi }+Q_{\eta \chi \xi }-Q_{\xi \eta \chi }).$$ (5)

Next, the superpotential is defined as


$${\mathbb{P}}_{\;\eta \chi }^{\tau }=-\frac{1}{2}{𝕃}_{\;\eta \chi }^{\tau }+\frac{1}{4}(Q^{\tau }-{\tilde{Q}}^{\tau })g_{\eta \chi }-\frac{1}{4}\delta^{\tau }{\;}_{(\eta }Q_{\chi )},$$ (6)

where


$$Q_{\tau }=Q_{\tau \;\eta }^{\;\eta },\;{\tilde{Q}}_{\tau }=Q_{\;\tau \eta }^{\eta }.$$ (7)

The non-metricity scalar *Q*, which characterizes the departure from metric compatibility, is defined as [63]


$$Q=-Q_{\tau \eta \chi }{\mathbb{P}}^{\tau \eta \chi }=-\frac{1}{4}(-Q^{\tau \chi \upsilon }Q_{\tau \chi \upsilon }+2Q^{\tau \chi \upsilon }Q_{\upsilon \tau \chi }-2Q^{\upsilon }{\tilde{Q}}_{\upsilon }+Q^{\upsilon }Q_{\upsilon }).$$ (8)

The total action in f(Q) gravity incorporates both matter ($L_{m}$) and electromagnetic ($L_{𝐞}$) Lagrangian terms, as shown in [43]


$$S=\int \frac{1}{2}f(Q)\sqrt{-g}d^{4}x+\int (L_{m}+L_{𝐞})\sqrt{-g}d^{4}x,$$ (9)

where


$$L_{𝐞}=-\frac{1}{16\pi }F_{\iota \sigma }F^{\iota \sigma }.$$ (10)

The electromagnetic field tensor $F_{\iota \sigma }$ is constructed from the antisymmetric derivative of the four-potential $\varphi_{\iota }$ as $F_{\iota \sigma }=\varphi_{\iota ,\sigma }-\varphi_{\sigma ,\iota }$. The gravitational field equations in f(Q) theory take the general form


$$\frac{-2}{\sqrt{-g}}\nabla_{\tau }(f_{Q}\sqrt{-g}{\mathbb{P}}_{\;\eta \chi }^{\tau })-\frac{1}{2}fg_{\eta \chi }-f_{Q}(P_{\eta \varpi \varsigma }Q_{\chi }^{\;\varpi \varsigma }-2Q_{\;\eta }^{\varpi \varsigma }P_{\varpi \varsigma \chi })=T_{\eta \chi }+E_{\eta \chi },$$ (11)

where $f_{Q}\equiv \frac{df(Q)}{dQ}$ represents the derivative of the gravitational function with respect to the non-metricity scalar. The energy-momentum tensor (EMT) encoding how electromagnetic fields are coupled to the spacetime curvature is given by [64]


$$E_{\eta \chi }=\frac{1}{4\pi }(F_{\eta }^{\sigma }F_{\chi \sigma }-\frac{1}{4}g_{\eta \chi }F_{\iota \sigma }F^{\iota \sigma }).$$ (12)

Maxwell’s equations mathematically establish the fundamental relationship between electric and magnetic fields, governing their mutual generation and interaction. In curved spacetime, Maxwell’s equations take the generally covariant form


$$(\sqrt{-g}F_{\eta \chi }{)}_{;\chi }=4\pi J_{\eta }\sqrt{-g},\;F_{\left[\eta \chi ;\delta \right]}=0,$$ (13)

where ξ denotes the charge density and $J_{\eta }=\xi u_{\eta }$ is the electric four-current with $u_{\eta }$ being the fluid’s four velocity.

The typical form of the spherically symmetric interior metric is as follows [65–69]


$$ds^{2}=-e^{\beta (r)}dt^{2}+e^{\alpha (r)}dr^{2}+r^{2}(d\theta^{2}+\sin^{2}\theta d\phi^{2}).$$ (14)

Goncalves and Lazzari [70] described the intensity of the electric field as


$$E(r)=4\pi \int_{0}^{r}\xi r^{2}e^{\alpha }\;dr.$$ (15)

where the charge contained within a sphere of radius *r* is represented by E(r. We may write the EMT for a isotropic perfect fluid as [71,72]


$$T_{\eta \chi }=(\rho +p)u_{\eta }u_{\chi }+pg_{\eta \chi },$$ (16)

where *p* is the pressure, and *ρ* is the fluid’s energy density. The components of the Einstein-Maxwell field equations in f(Q) gravity are written as follows


$$\rho +\frac{q^{2}}{r^{4}}=\frac{f}{2}+\frac{2}{r}f_{QQ}e^{-\alpha }Q^{\prime }-f_{Q}\{Q+\frac{e^{-\alpha }}{r}(\beta^{\prime }+\alpha^{\prime })+\frac{1}{r^{2}}\},$$ (17)

$$p-\frac{q^{2}}{r^{4}}=-\frac{f}{2}+f_{Q}(Q+\frac{1}{r^{2}}),$$ (18)

$$p+\frac{q^{2}}{r^{4}}=f_{Q}[\frac{Q}{2}-e^{-\alpha }\{\frac{\beta^{\prime \prime }}{2}+(\frac{\beta^{\prime }}{4}+\frac{1}{2r})(\beta^{\prime }-\alpha^{\prime })\}]-f_{QQ}e^{-\alpha }Q^{\prime }(\frac{\beta^{\prime }}{2}+\frac{1}{r})-\frac{f}{2},$$ (19)

$$0=\frac{1}{2}\cot\theta Q^{\prime }f_{QQ}.$$ (20)

According to [73], the non-metricity scalar is provided as


$$Q=-\frac{2e^{-\alpha (r)}}{r}(\beta^{\prime }(r)+\frac{1}{r}),$$ (21)

where $r=\frac{d}{dr}$. The traditional definition of the linear model for f(Q) theoretical framework is obtained by Eq (20) to solve the analogous field equations. This is given by [74]


$$f_{QQ}(Q)=0\;⟹\;f_{Q}(Q)=\lambda \;⟹\;f(Q)=\lambda Q+\gamma ,$$ (22)

with *λ* and *γ* being real-valued constants. This straightforward functional form greatly simplifies the mathematical derivation of solutions and allows for controlled departures from GR. The field equations resulting from f(Q) gravity become identical to those of GR under this assumption, where non-metricity disappears and the metric alone explains the curvature of spacetime. We have multiple reasons for using the linear model: theoretical coherence and the unique astrophysical setting of compact stars.

- Our method is based on the “geometric trinity of gravity”, which holds that curvature (GR), torsion (TEGR), or non-metricity (STEGR) can all be used to define GR in an analogous way. While these formulations are dynamically equivalent in their basic forms, they are *geometrically distinct*. The f(Q) gravity framework is built upon the STEGR formulation, where gravity is attributed to non-metricity Q in a flat, torsion-free geometry. The choice of the linear f(Q) model allows us to study compact stars within this specific geometric paradigm.
- Crucially, the field equations in f(Q) gravity are typically derived in the so-called “coincident gauge”, where the affine connection is trivial. This gauge selection reflects a particular physical arrangement within the symmetric teleparallel geometry and is not only a mathematical convenience. The resulting solutions are interpreted within the framework of this gauge, which differs from the Riemannian geometry of standard GR both conceptually and physically, even for the linear model. As a result, our solutions characterize compact stars in a spacetime where gravity is only expressed by flat geometry’s non-metricity rather than curvature. Even though the dynamical equations are theoretically comparable to GR for a particular set of parameters, this offers a fresh geometric viewpoint on stellar structure.
- Investigating how new physics appears as departures from GR is one of modified gravity’s main objectives. The most logical and theoretically sound place to start such an analysis is with the linear model (22). It serves as a minimal extension with two key features
  – The effective gravitational constant is rescaled by the parameter *λ*. We methodically examine the effects of a strengthening (λ<−1) or weakening (λ>−1) of the gravitational interaction within the non-metricity framework on the structure and stability of compact stars by treating *λ* as a free parameter distinct from −1. This parameter space is tested by our investigation of stars such as LMC X-4 and Her X-1, investigating regimes that are not only comparable to GR.
  – An effective cosmological constant is represented by the constant *γ*. This term adds to the energy-momentum tensor in the high-density environment of a compact star, thereby changing the EoS. We clearly show the effects of its real alteration on the mass-radius relation, pressure, and density profiles.

We are not researching GR, but rather a two-parameter family of theories that incorporate GR as a single, specific point (λ=−1, γ=0) [73], by examining the physical acceptability of our solutions throughout a range of (λ,γ values. Our findings show that this extended theory contains a class of physically feasible stellar solutions.

- A substantial amount of literature on f(Q) gravity, such as [75–83], which also used the linear model to develop innovative solutions, substantially supports our methodological choice. Specifically, Solanki et al. [84] solved the modified field equations within this framework, obtaining promising results. Furthermore, Zhao [85] explored its application to two pivotal scenarios: static spherically symmetric spacetimes and homogeneous, isotropic cosmological models. Within this theoretical framework, Wang et al. [86] discovered stable solutions for various compact stars, while Maurya et al. [87] generated anisotropic solutions characterized by vanishing complexity and evaluated their physical viability. Furthermore, Bhar et al. [88] analyzed the physical properties and maximum allowable mass of a hybrid star candidate. This agreement shows that, before moving on to more intricate, non-linear forms, the linear model is widely acknowledged as the fundamental and most manageable form for obtaining new analytic solutions in f(Q) gravity.
- A solid starting point is to first analyze the system with a linear model. This gives us a clear baseline for how compact stars behave under symmetric teleparallel gravity. Once that foundation is in place, extending to non-linear f(Q) terms (which will deviate more sharply from GR) becomes straightforward. Skipping the linear benchmark, though, would leave any later non-linear findings open to misinterpretation.

Conclusively, our work is not a misunderstanding but a deliberate exploration of compact stars within the geometric framework of f(Q) gravity. Substituting Eqs (21) and (22) into the system (17)-(19) yields


$$\begin{array}{cc} \rho +\frac{q^{2}}{r^{4}} & \;=\frac{-2\lambda +\gamma r^{2}-2\lambda e^{-\alpha (r)}\left(r\alpha^{\prime }(r)-1\right)}{2r^{2}}, \end{array}$$ (23)

$$\begin{array}{cc} p-\frac{q^{2}}{r^{4}} & \;=-\frac{e^{-\alpha (r)}}{8r^{2}}(8\lambda -\lambda r^{2}\alpha^{\prime }(r)\beta^{\prime }(r)+6\gamma r^{2}e^{\alpha (r)}+2\lambda r^{2}\beta^{\prime \prime }(r)+\lambda r^{2}\beta^{\prime }(r{)}^{2}\\ & \;-2\lambda r\alpha^{\prime }(r)-8\lambda e^{\alpha (r)}+10\lambda r\beta^{\prime }(r)), \end{array}$$ (24)

$$\begin{array}{cc} p+\frac{q^{2}}{r^{4}} & \;=\frac{e^{-\alpha (r)}\left(-\lambda \left(r\beta^{\prime }(r)+2\right)\left(\beta^{\prime }(r)-\alpha^{\prime }(r)\right)-2\gamma re^{\alpha (r)}-2\lambda r\beta^{\prime \prime }(r)\right)}{4r}. \end{array}$$ (25)

After solving the above system of equations simultaneously, we get the following expressions


$$\begin{array}{cc} \rho & \;=\frac{\gamma }{4}-\frac{1}{8}\lambda e^{-\alpha (r)}\alpha^{\prime }(r)\beta^{\prime }(r)-\frac{5\lambda e^{-\alpha (r)}\alpha^{\prime }(r)}{4r}+\frac{1}{4}\lambda e^{-\alpha (r)}\beta^{\prime \prime }(r)+\frac{1}{8}\lambda e^{-\alpha (r)}\beta^{\prime }(r{)}^{2}\\ & \;-\frac{3\lambda e^{-\alpha (r)}\beta^{\prime }(r)}{4r}, \end{array}$$ (26)

$$\begin{array}{cc} p & \;=-\frac{e^{-\alpha (r)}}{8r^{2}}(8\lambda -\lambda r^{2}\alpha^{\prime }(r)\beta^{\prime }(r)+6\gamma r^{2}e^{\alpha (r)}+2\lambda r^{2}\beta^{\prime \prime }(r)+\lambda r^{2}\beta^{\prime }(r{)}^{2}-2\lambda r\alpha^{\prime }(r)\\ & \;-8\lambda e^{\alpha (r)}+10\lambda r\beta^{\prime }(r)), \end{array}$$ (27)

$$\begin{array}{cc} q^{2} & \;=\frac{1}{8}r^{2}e^{-\alpha (r)}(8\lambda +\lambda r^{2}\alpha^{\prime }(r)\beta^{\prime }(r)+2\gamma r^{2}e^{\alpha (r)}-2\lambda r^{2}\beta^{\prime \prime }(r)-\lambda r^{2}\beta^{\prime }(r{)}^{2}+2\lambda r\alpha^{\prime }(r)\\ & \;-8\lambda e^{\alpha (r)}+6\lambda r\beta^{\prime }(r)). \end{array}$$ (28)

## 3 Development of novel analytical interior geometries

Scientists have continuously strived to find solutions to Einstein’s field equations and their various modified forms. Having identified this critical challenge, researchers must now explore potential solutions. While multiple approaches exist to address this issue, our focus will be on leveraging two different metric ansatz, which have demonstrated efficacy in similar contexts in GR. Each proposed solution will be thoroughly examined in the following subsections.

### 3.1 Stellar model 1

As evident from Eqs (26) and (27), this system remains under-determined due to the presence of four unknown variables. One potential approach to resolve this system involves treating any two unknowns as free parameters, although this methodology has received limited acceptance in the existing research. The alternative approach requires simultaneous consideration of two distinct constraints within the system. Accordingly, we commence our investigation by implementing a widely accepted ansatz. The physical viability of anisotropic solutions derived from this metric has successfully been established by Murad and Fatema [89]. In related studies, this metric has been employed to analyze the structure of compact stars, and the analyses verified that the resulting stellar configurations are both physically stable and singularity-free [90–92]. These metric components adopt the functional forms proposed in [93]


$$e^{\alpha (r)}=\frac{X\left(2r^{2}+Y\right)}{\left(X-r^{2}\right)\left(r^{2}+Y\right)},\;e^{\beta (r)}=Z\left(\frac{r^{2}}{Y}+1\right).$$ (29)

To maintain dimensional consistency with the dimensionless metric components, the constants *X* and *Y* must possess dimensions of $\ell^{2}$, while *Z* must remain dimensionless. These constants will be established through the application of appropriate matching conditions in subsequent analysis. Substituting the functional forms of $e^{\beta (r)}$ and $e^{\alpha (r)}$ into Eqs (26) and (27) yields


$$\rho =\frac{4r^{4}(\gamma X-3\lambda )+2r^{2}(-6\lambda X+2\gamma XY-9\lambda Y)+Y(-14\lambda X+\gamma XY-10\lambda Y)}{4X{\left(2r^{2}+Y\right)}^{2}},$$ (30)

$$p=-\frac{3\left(2r^{2}(\gamma X-3\lambda )+2\lambda X+\gamma XY-2\lambda Y\right)}{4X\left(2r^{2}+Y\right)}.$$ (31)

Darmois junction conditions serve as fundamental constraints in relativistic astrophysics, providing the mathematical framework for consistently matching both field variables and their spatial derivatives across interface boundaries between different matter configurations or spacetime geometries. The matching conditions, comprising both metric tensor’s continuity and extrinsic curvature’s continuity, guarantee smooth geometric matching of spacetime manifolds and self-consistent solutions of the field equations across boundary interfaces. These matching conditions serve three essential purposes: *(i)* they provide critical constraints for solving boundary value problems, *(ii)* they govern wave propagation characteristics across discontinuous media, and *(iii)* they determine the dynamical evolution of interfaces in relativistic systems [94]. To establish continuity between the interior solution (given by metric (18)) and the exterior spacetime, we consider the Reissner-Nordström metric as the latter geometry. The line element for this metric is given as


$$ds^{2}=-(1-\frac{2M}{r}+\frac{Q^{2}}{r^{2}})dt^{2}+(1-\frac{2M}{r}+\frac{Q^{2}}{r^{2}}{)}^{-1}dr^{2}+r^{2}(d\theta^{2}+\sin^{2}\theta d\phi^{2}),$$ (32)

with the total mass *M* and electric charge *Q* of the stellar configuration. The continuity of intrinsic geometry at the boundary surface ensures the necessary matching condition, requiring all metric components to remain continuous across the interface. The second fundamental form governs the matching conditions for extrinsic curvature at the boundary interface, ensuring the proper embedding of the hypersurface in adjacent spacetime regions. This constraint necessarily requires the pressure to become zero (p(R)=0) at the star’s surface r=R, with *R* representing the radius. These conditions generate three fundamental mathematical relations


$$1-\frac{2M}{R}+\frac{Q^{2}}{R^{2}}=\frac{\left(X-R^{2}\right)\left(R^{2}+Y\right)}{X\left(2R^{2}+Y\right)},$$ (33)

$$1-\frac{2M}{R}+\frac{Q^{2}}{R^{2}}=Z\left(\frac{R^{2}}{Y}+1\right),$$ (34)

$$p(R)=0=-3\left(2R^{2}(\gamma X-3\lambda )+2\lambda X+\gamma XY-2\lambda Y\right).$$ (35)

Notably, in many extensions of GR, such as higher-order theories like f(R) gravity, the presence of additional degrees of freedom or higher-derivative terms can lead to modified boundary conditions at the interface between interior and exterior spacetimes. However, in the specific framework of f(Q) gravity adopted in our work, the standard Darmois junction conditions remain valid for the following reasons. First, f(Q) gravity is constructed as a second-order theory in its field equations, similar to GR, rather than introducing fourth-order or higher derivatives as seen in curvature-based modifications like f(R) theory. The action in f(Q) is varied with respect to the metric tensor and an independent affine connection, but in the symmetric teleparallel formulation, we employ the coincident gauge where the affine connection vanishes. In this gauge, which is commonly used for spherically symmetric solutions and is implicitly adopted in our derivations, all geometric information including non-metricity is fully encoded in the metric and its first derivatives. The non-metricity tensor reduces to $-g_{\mu \nu ,\lambda }$, ensuring that *Q* is a direct function of the metric derivatives without independent connection terms at the boundary. Consequently, the junction conditions derive from the same principles as in GR: the requirement for a smooth embedding of the time-like hypersurface (the stellar surface at r=R) into the full spacetime manifold. By solving Eqs (33)-(35) simultaneously, we derive explicit analytical solutions for the parameter set (X,Y,Z) as


$$X=\frac{4\lambda R^{4}}{4\lambda MR-2\lambda Q^{2}+\gamma R^{4}},$$ (36)

$$Y=-\frac{R^{2}\left(4\lambda R(R-3M)+6\lambda Q^{2}+\gamma R^{4}\right)}{-4\lambda MR+2\lambda Q^{2}+\gamma R^{4}},$$ (37)

$$Z=\frac{6\lambda \left(Q^{2}-2MR\right)+\gamma R^{4}}{4\lambda R^{2}}+1.$$ (38)

The derived constants will be utilized in the following graphical investigation to analyze the physical characteristics of the obtained solutions. The incorporation of modified parameters *λ* and *γ* within these constants facilitates a controlled investigation of f(Q) gravity’s influence on the physical properties of compact stellar objects.

### 3.2 Stellar model 2

Our investigation employs another novel metric ansatz to assess the solution’s viability in describing observed compact astrophysical systems. The metric adopted in our current study has demonstrated both theoretical consistency and physical viability within GR, having successfully generated physically plausible solutions for compact astrophysical systems in prior research. In this subsection, we critically analyze the validity of the chosen metric ansatz within the framework of modified f(Q) gravity. The Kohler-Chao-Tikekar metric offers a foundational framework for modeling realistic, spherical stellar objects, rendering it a subject of extensive study in astrophysics [95,96]. Consequently, the accurate representation of pressure anisotropy is a fundamental requirement for any model attempting to characterize matter in ultra-dense stellar environments. The versatility of the Kohler-Chao-Tikekar metric is evidenced by its successful application across diverse contexts. For instance, Maurya et al. [97] employed it to derive physically acceptable charged isotropic solutions. Albalahi et al. [98] extended this model, using gravitational decoupling to generate anisotropic solutions from the isotropic seed source under the condition of vanishing complexity. In a similar approach, Siza et al. [99] applied the same ansatz with a complexity-free constraint to develop models for ultra-relativistic stellar systems. Some other interesting works have also been done in the literature using this metric [100,101]. This is given by


$$e^{\alpha (r)}=\frac{a+2br^{2}}{a+br^{2}},\;e^{\beta (r)}=a+br^{2},$$ (39)

with constants *b* and *a* having dimensions of $\frac{1}{\ell^{2}}$ and null, respectively. When $e^{\alpha (r)}$ and $e^{\beta (r)}$ are substituted in Eqs (26) and (27), we get


$$\rho =\frac{a^{2}\gamma +2ab\left(2\gamma r^{2}-7\lambda \right)+4b^{2}r^{2}\left(\gamma r^{2}-3\lambda \right)}{4{\left(a+2br^{2}\right)}^{2}},$$ (40)

$$p=-\frac{3\left(a\gamma +2b\left(\lambda +\gamma r^{2}\right)\right)}{4\left(a+2br^{2}\right)}.$$ (41)

To employ the Darmois junction conditions at the stellar boundary, the exterior spacetime metric of a static, spherically symmetric star must fulfill two criteria: *(i)* dependence solely on the radial coordinate *r*, and *(ii)* asymptotic flatness. Consistent with these constraints, we select the Schwarzschild vacuum solution (given by Eq (32)) to describe the exterior spacetime. At the stellar surface r=R, we have a set of following equations


$$1-\frac{2M}{R}+\frac{Q^{2}}{R^{2}}=\frac{a+bR^{2}}{a+2bR^{2}},$$ (42)

$$1-\frac{2M}{R}+\frac{Q^{2}}{R^{2}}=a+bR^{2},$$ (43)

$$p(R)=0=-3\left(a\gamma +2b\left(\lambda +\gamma R^{2}\right)\right),$$ (44)

from which we uniquely determine the three constants as


$$a=\frac{-4MR+2Q^{2}+R^{2}}{R^{2}},$$ (45)

$$b=-\frac{Q^{2}-2MR}{R^{4}},$$ (46)

$$\lambda =\frac{\gamma R^{4}}{2Q^{2}-4MR}.$$ (47)

We stress that the constants (a,b,λ) are dependent on *γ*, which serves as an unconstrained free parameter in our framework. These values will be utilized in our following investigation to assess the physical admissibility of the solution presented in Eqs (40) and (41).

## 4 Physical interpretation of the obtained models

This section presents a comprehensive evaluation of the solution’s physical validity through three key assessments: *(i)* geometric compatibility, *(ii)* matter configuration’s characteristics, and *(iii)* thermodynamic equilibrium conditions. This comprehensive validation guarantees that our solutions both accurately represent compact astrophysical objects and satisfy all essential theoretical and observational requirements. This investigation systematically assesses the solutions’ stability against physical perturbations, examining their behavior under extreme astrophysical conditions and evaluating their suitability as realistic stellar configurations. The initial phase of our investigation establishes two fundamental viability criteria: geometric regularity of all metric components throughout the spacetime manifold, and physically meaningful behavior of matter field variables, both being essential prerequisites for astrophysical relevance. The following subsections provide comprehensive analysis of these parameters and quantitative assessment frameworks employed to systematically examine these characteristics. Notably, we use a range of model parameters as λ=−0.9 and γ=0,0.001,0.002,0.003,0.004,0.005 and charge Q=0.5,1.5. We also use the star candidate LMC X-4, which has a mass of $M=1.04\pm 0.09M_{⨀}$, and a radius of R=8.301±0.2km [102] to examine how the modified f(Q) theory affects the presence of stellar structures. Further, the interior charge is also an unknown quantity, and to analyze our solutions graphically, we assume it to be $q=\vartheta r^{3}$ (with *ϑ* being a constant having the dimension of $\ell^{-2}$). Multiple values of this constant have been considered, however, ϑ=0.001 provides us the suitable results [103,104].

In the context of compact stars, the inclusion of electric charge serves as a theoretical tool to explore deviations from neutrality, which can arise from various physical mechanisms in extreme environments. While neutron stars and other compact objects are generally expected to be electrically neutral on macroscopic scales due to the high conductivity of degenerate matter and the presence of surrounding plasma that facilitates charge neutralization, small net charges can theoretically persist or be induced under certain conditions. For instance, in the dense interiors of neutron stars, asymmetries in the distribution of charged particles (such as protons, electrons, and muons) due to strong magnetic fields, rapid rotation, or phase transitions could lead to localized charge imbalances. Additionally, during the formation process via supernova explosions or accretion in binary systems like LMC X-4, transient charge separations might occur, although these are typically damped by electromagnetic interactions. In modified gravity theories such as f(Q), the non-metricity introduces additional degrees of freedom that can couple to electromagnetic fields, potentially amplifying or stabilizing such charges on astrophysical scales.

Regarding the specific values of the exterior charge adopted in our analysis, these are chosen as illustrative parameters to probe the sensitivity of the stellar structure to electromagnetic contributions within the f(Q) framework. For a compact star like LMC X-4, which is an accreting neutron star, astrophysical realism requires that any net charge be extremely small to avoid observational contradictions. In standard GR, theoretical upper limits on the net charge of neutron stars are stringent: for instance, charges exceeding $\sim 10^{18}C$ would lead to significant electromagnetic repulsion that could disrupt the star’s stability or produce detectable electromagnetic signatures, such as enhanced X-ray emissions or altered pulsar timing. Observational constraints from X-ray binaries like LMC X-4, based on data from telescopes such as Chandra and XMM-Newton, indicate no evidence of large-scale charge imbalances, with upper limits on surface electric fields below $\sim 10^{12}V/m$ to avoid excessive pair production or radiation. Furthermore, gravitational wave observations from events like GW170817 constrain exotic electromagnetic effects in neutron star mergers, implying that charges must be $<10^{-10}$ times the mass in geometric units (Q/M≪1) to match the inferred tidal deformabilities and post-merger remnants.

### 4.1 Metric elements and fluid variables

**Fig 1:**
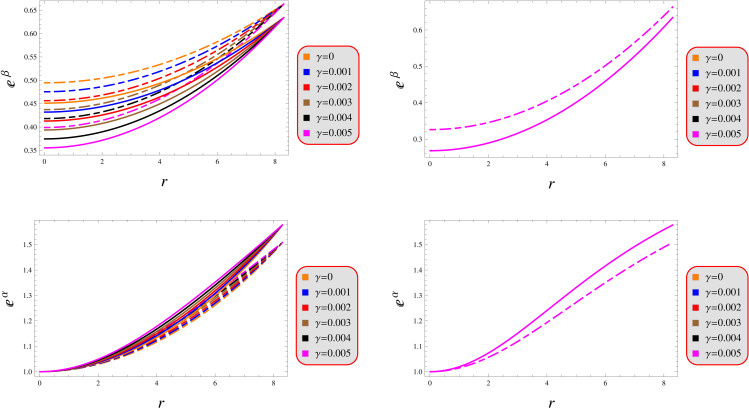
Geometric functions under models 1 (left) and 2 (right) for *Q* = 0.5 (solid) and 1.5 (dashed).

The metric tensor components at the stellar core must obey strict regularity constraints: *(i)* the radial component must normalize to unity at the center, *(ii)* the temporal component must maintain finite constant value between 0 and 1, and *(iii)* both components must demonstrate strictly monotonically increasing radial dependence outwards. Furthermore, these metric components must maintain strictly positive and finite values across the entire spatial domain of interest. For physical consistency at the stellar core, all first-order radial derivatives of the metric potentials vanish identically at r=0. Fig 1 graphically displays all four elements, demonstrating their strictly positive values, and physically consistent radial evolution throughout the domain.

**Fig 2:**
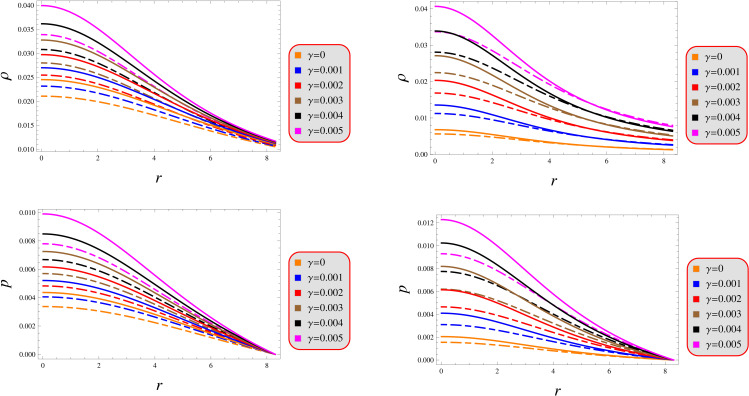
Fluid determinants under models 1 (left) and 2 (right) for *Q* = 0.5 (solid) and 1.5 (dashed).

A thorough analysis of the fluid variables’ profile provides critical insight into both the structural configuration and dynamic stability of the stellar system. The energy density must satisfy three key physical requirements: *(i)* strict positivity, *(ii)* finiteness everywhere, and *(iii)* monotonic decrease outward. These requirements prevent exotic matter, eliminate singularities, and maintain gravitational confinement in relativistic stars. These rules ensure the pressure stays physically realistic: highest at the star’s core, gradually decreasing outward, and dropping to zero at the surface – matching how compact objects behave in nature. Fig 2 graphically demonstrates the radial profile of these physical quantities, exhibiting behavior fully consistent with the theoretical requirements outlined previously. Our analysis reveals an inverse correlation between energy density and the parameters (λ,γ), yielding comparatively denser structures in GR than in the modified gravity framework. Comparative analysis reveals that solution 2 generates significantly denser stellar configurations than solution 1. Both solutions exhibit identical pressure profiles, with its vanishing at the surface boundary. The graphs in Fig 2 consistently demonstrate this behavior, providing visual confirmation of our analytical results.

Determining a solution’s physical plausibility requires evaluating its radial derivative profiles, which characterize the spatial variation of stellar material properties. These gradients map how thermodynamic quantities transition between different layers of the star’s internal structure. They can be stated mathematically as


$${\left(\frac{d\rho }{dr}\right)}_{r=0}=0={\left(\frac{dp}{dr}\right)}_{r=0},\;{\left(\frac{d^{2}\rho }{dr^{2}}\right)}_{r=0}<0,\;{\left(\frac{d^{2}p}{dr^{2}}\right)}_{r=0}<0.$$

**Fig 3:**
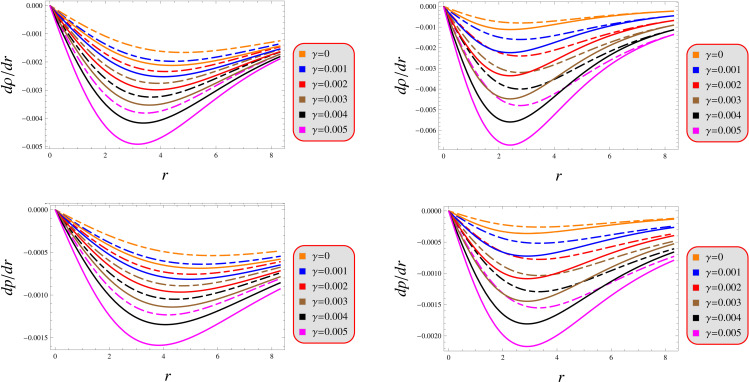
Gradient under models 1 (left) and 2 (right) for *Q* = 0.5 (solid) and 1.5 (dashed).

As illustrated in Fig 3, both proposed solutions demonstrate satisfying the criteria through their pressure distributions while maintaining energetically viable density profiles that adhere to fundamental stellar requirements. While these solutions remain valid within the considered parameters range, graphical representation for the second-order derivatives is intentionally omitted from this analysis to maintain focus on theoretical boundaries.

### 4.2 Equation of state

**Fig 4:**
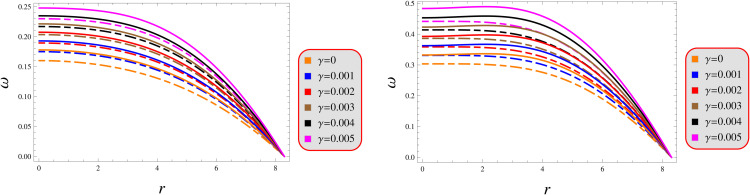
Equation of state parameter under models 1 (left) and 2 (right) for *Q* = 0.5 (solid) and 1.5 (dashed).Equation of state parameter under models 1 (left) and 2 (right) for *Q* = 0.5 (solid) and 1.5 (dashed).

The barotropic equation of state is defined as p=ωρ, where Ω is a constrained parameter. The expression of this component is represented as $\omega =\frac{p}{\rho }$. For physical viability, the ratio of pressure to density must fulfill the criteria [0,1]. The solution maintains thermodynamic validity by satisfying all fundamental requirements of stellar structure’s physics. As evidenced in Fig 4, the numerical results verify stable parameter preservation across the entire stellar model. The observed radial profiles exhibit characteristic monotonic decay of material properties, confirming density-driven thermodynamic behavior throughout the system’s configuration.

### 4.3 Mass, compactness, and surface redshift

The total gravitational mass enclosed within a spherical region of radius *r* can be mathematically expressed through the volume integral


$$m(r)=\frac{1}{2}\int_{0}^{r}r^{2}\rho (r)dr\;⟹\;m^{\prime }(r)=\frac{1}{2}r^{2}\rho (r).$$ (48)

**Fig 5:**
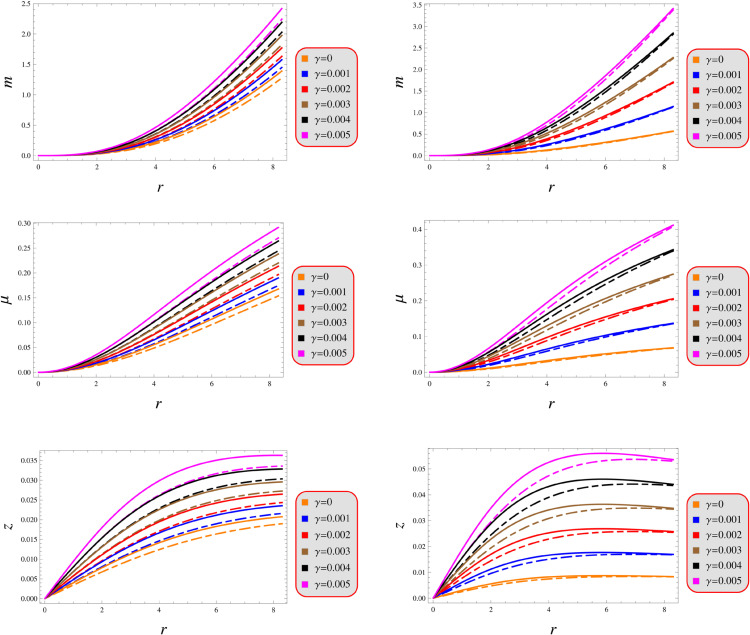
Physical quantities under models 1 (left) and 2 (right) for *Q* = 0.5 (solid) and 1.5 (dashed).

The differential equation admits both analytical and numerical approaches, with the choice of method being determined by the functional complexity of the energy density. Fig 5 (top plots), displays the behavior of the mass function, demonstrating that m(r asymptotically approaches zero as the radial coordinate *r* is zero. Alternatively, the function exhibits strictly increasing radial dependence, with mass consistently growing at larger distances from the origin.

The compactness parameter, defined as the ratio of gravitational mass to circumferential radius, governs both the structural stability and gravitational confinement of astrophysical objects. Relativistic compact objects such as neutron stars demonstrate pronounced spacetime curvature, manifesting strong-field gravitational phenomena not observed in Newtonian systems. According to Buchdahl [105], a viable compact structure needs a dimensionless compactness factor $\left(\mu (r)=\frac{m(r)}{r}\right)$ less than 0.44. While this theoretical bound was initially derived for idealized isotropic systems with perfect spherical symmetry, subsequent research has demonstrated its potential applicability to isotropic matter configurations as well. Photons propagating outward through a strong gravitational field experience an energy deficit, manifesting as a wavelength shift towards the red spectrum. This gravitational redshift phenomenon serves as both a diagnostic tool for stellar compactness and a robust test of relativistic predictions. This is determined by


$$z=\{1-2\mu (r){\}}^{-\frac{1}{2}}-1.$$ (49)

The gravitational redshift within compact stellar interiors scales linearly with the object’s compactness parameter, showing systematic enhancement as the strength of gravitational confinement increases. In the case of isotropic matter configurations, theoretical analysis established an absolute maximum surface redshift value of z=5.211 at the object’s exterior boundary. This fundamental limit serves as a crucial viability criterion for physically admissible solutions [106]. Fig 5 graphically presents both components, demonstrating their well-behaved radial evolution throughout the complete spatial extent of the system.

### 4.4 Moment of inertia

The moment of inertia serves as a fundamental diagnostic tool for characterizing rotational dynamics in compact stellar objects. Its quantitative analysis reveals: The internal mass distribution’s role in governing rotational behavior, a definitive relationship between static structural parameters and dynamic rotational properties and a maximum value occurring in the slow-rotation limit for given mass-radius relations. The mathematical expression is [107]


$$I=\frac{2}{5}\left[\left(1+\frac{M}{R}\right)MR^{2}\right].$$ (50)

**Fig 6:**
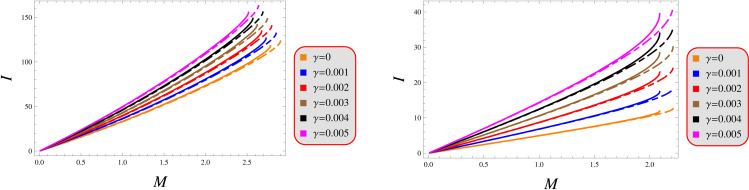
Moment of inertia under models 1 (left) and 2 (right) for *Q* = 0.5 (solid) and 1.5 (dashed).

Our investigation establishes a monotonic dependence between gravitational mass and rotational inertia, with quantitative analysis confirming their near-linear scaling across the parameter space of compact objects. Fig 6 demonstrates a consistent mass-dependent enhancement of rotational inertia, revealing a strictly monotonically increasing relationship between these fundamental parameters. This characteristic behavior originates from the progressive radial expansion of matter distribution relative to the rotational axis as gravitational mass increases. The moment of inertia asymptotically converges to a mass-specific saturation value, marking the onset of structural stability boundaries. When exceeding this threshold configuration, further mass accumulation produces diminishing returns in modifying the object’s rotational characteristics.

### 4.5 Viability conditions

Physically realistic stellar configurations must satisfy fundamental constraints on their internal energy-momentum distribution. These viability criteria, known as the energy conditions, comprise five principal categories in GR: trace, dominant, strong, null, and weak bounds. These conditions serve as critical diagnostics for characterizing the solution’s matter content, distinguishing between conventional baryonic matter and exotic field configurations. These constraints establish rigorous bounds on permissible pressure-energy density parameter spaces, enabling researchers to validate theoretical predictions against astrophysical observations. They are defined as follows


$$\rho +\frac{q^{2}}{r^{4}}\geq 0,\;\rho +p\geq 0,\;\rho +3p+\frac{2q^{2}}{r^{4}}\geq 0,\;\rho -3p\geq 0,\;\rho -p+\frac{2q^{2}}{r^{4}}\geq 0.$$

**Fig 7:**
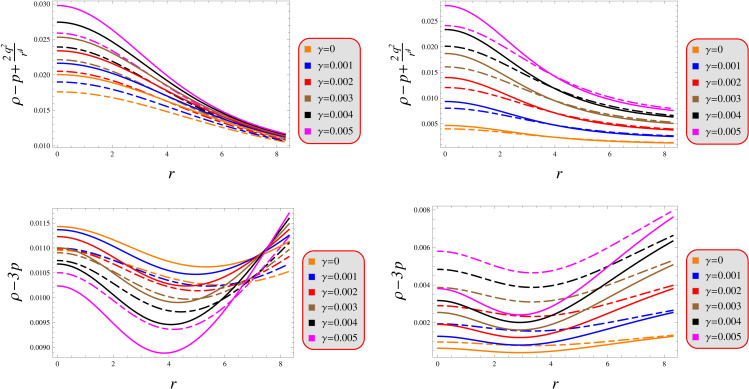
Viability under models 1 (left) and 2 (right) for Q = 0.5 (solid) and 1.5 (dashed).

The consistently positive values displayed in Fig 2 suggest physical plausibility, from which we rigorously confirm: *(i)* the weak (ρ+p≥0, and *(ii)* the strong energy condition (ρ+3p≥0. Fig 7 visually exhibit the remaining conditions corresponding to solutions 1 and 2 whose strictly positive-definite nature throughout the stellar configuration confirms the exclusive presence of conventional baryonic matter, thereby establishing the solutions’ thermodynamic consistency and physical viability.

## 5 Stability analysis

Stability assessment of compact stellar solutions represents a fundamental component of relativistic astrophysics, serving as the critical determinant for both their physical admissibility and capacity to model observable phenomena. This methodology systematically examines hydrostatic equilibrium states in degenerate compact objects (including both neutron stars and white dwarfs), while rigorously assessing their dynamic response to spherically symmetric perturbations. Our formalism establishes exact stability criteria through analytic derivation of the critical gravitational binding energy that delineates: stable hydrostatic equilibrium configurations and collapse-prone unstable regimes. Several of these diagnostic methods will be systematically applied in the following subsections to evaluate different stability conditions.

### 5.1 Hydrostatic equilibrium

**Fig 8:**
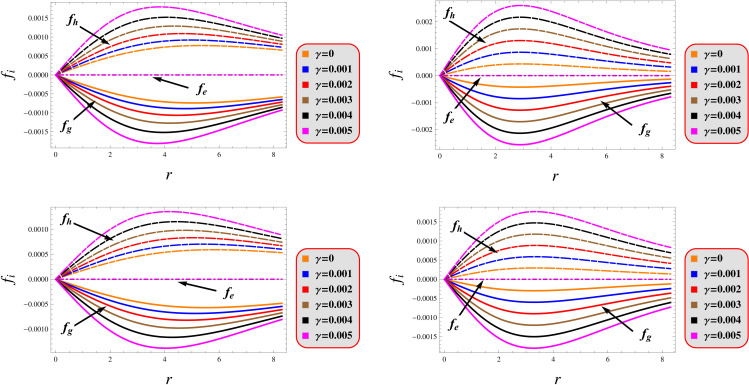
Equilibrium of forces under models 1 (left) and 2 (right) for *Q* = 0.5 (upper) and 1.5 (lower).

This investigation systematically examines the equilibrium properties of relativistic stellar configurations, accounting for the competing interplay of fundamental forces governing their structure. In the study of stellar structures, hydrostatic equilibrium describes a stable configuration wherein the compressive force of self-gravity is exactly balanced by expansive forces arising from both thermal and quantum mechanical pressure contributions. The mechanical equilibrium of dense stellar remnants is supported by quantum degeneracy effects – relativistic electrons opposing gravitational collapse in white dwarfs, while neutron stars rely on nucleon degeneracy augmented by strong-force repulsion at extreme densities. The Tolman-Oppenheimer-Volkoff formalism constitutes the definitive general relativistic description of hydrostatic equilibrium in strongly self-gravitating systems, governing the pressure-mass relation for compact objects within Einstein’s field equations. This equilibrium condition emerges from the exact cancelation of compressive gravitational forces by outward pressure. This equation takes the following form [93,108]


$$f_{\text{total}}\equiv f_{g}+f_{h}+f_{e}=0,$$ (51)

where $f_{g}=-\frac{1}{2}(p+\rho )\beta^{\prime }(r),\;f_{h}=-p^{\prime },\;f_{e}=\frac{2qq^{\prime }}{r^{4}}$. Fig 8 contains these three forces. The graphical analysis demonstrates exact force equilibrium across all radial positions, confirming that the proposed configurations satisfy hydrostatic balance universally throughout their parameter space. These results provide empirical validation for the charged compact object solutions within the modified gravity paradigm, demonstrating their consistency with astrophysical observations.

### 5.2 Causality condition

**Fig 9:**
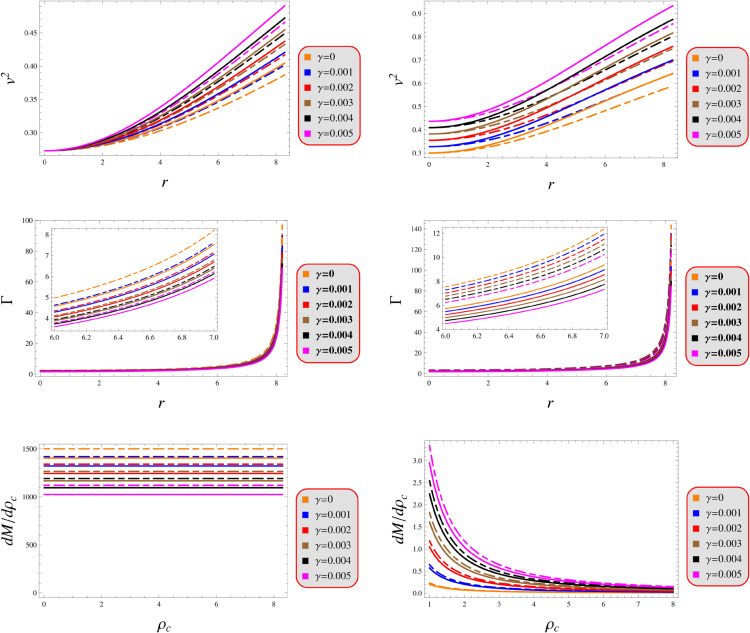
Stability under models 1 (left) and 2 (right) for *Q* = 0.5 (solid) and 1.5 (dashed).

The universal speed limit, embedded in the causal structure of spacetime through Einstein’s relativity, prohibits any physical signal or particle from exceeding light speed in vacuum. Relativistic causality imposes strict upper limits on mechanical wave propagation speeds within compact objects, mandating that the sound speed must satisfy at all interior locations [109]. This fundamental constraint ensures both causal propagation and dynamic stability throughout the stellar configuration. This is mathematically expressed $0\leq v^{2}=\frac{dp}{d\rho }\leq 1$. Fig 9 demonstrates strict adherence to this causality condition for both solutions, with all parameter variations satisfying the required bounds.

### 5.3 Adiabatic index

The adiabatic index represents a fundamental measure of stellar material stiffness, characterizing how a compressed fluid element resists density changes without energy exchange. For compact objects, this parameter crucially determines their stability against gravitational collapse. Stellar stability fundamentally requires the adiabatic index to exceed the critical threshold of $\frac{4}{3}$ [110]. When the adiabatic index falls below the critical $\frac{4}{3}$ threshold, gravitational compression dominates over pressure restoration forces, potentially triggering loss of hydrostatic equilibrium and subsequent stellar collapse. This element manifests as $\Gamma =\frac{\rho +p}{p}\frac{dp}{d\rho }$. Fig 9 demonstrates that both solutions satisfy this stability criterion, as evidenced by a complete compliance with the required parametric bounds.

### 5.4 Harrison-Zeldovich-Novikov’s criteria

The eigen-frequencies of fundamental modes were initially calculated by Chandrasekhar [111]. Later, Harrison et al. [112] performed analogous computations, confirming and expanding upon these findings. Zeldovich and Novikov [113] later refined these computational methods, enhancing the efficiency of the framework originally established by Harrison and his colleagues. To establish this relationship, they proposed that the adiabatic index for quasi-static matter deformation resembles that of an oscillating stellar object [113]. From this theoretical framework, they established a stability condition for equilibrium states: the system remains stable only when the total mass increases with central density $(dM/d\rho_{c}>0$. Conversely, configurations become unstable when $dM/d\rho_{c}<0$. The numerical analysis confirms a positive-definite values of $dM/d\rho_{c}$ across all radial positions for both stellar models, with Fig 9 (last row) providing explicit verification of this monotonic relationship. These results demonstrate that our models fully comply with the Harrison-Zeldovich-Novikov stability condition. Furthermore, independent verification comes from recent numerical studies [114,115] which corroborate our findings through complementary methodologies.

## 6 Concluding remarks

Our investigation of the charged f(Q) gravity has yielded two exact closed-form solutions characterizing perfect fluid spheres admitting a spherical symmetry. The analytical procedure comprised two sequential phases: *(i)* establishment of the isotropic pressure condition, followed by *(ii)* the derivation of the generalized field equations for static perfectly spherical gravitational object. The solution process required imposing an additional constraint, as we identified the system of two independent field equations as under-determined, involving four unknown functions (metric coefficients and matter variables). To ensure physically meaningful solutions, we implemented two well-behaved spacetimes, both exhibiting non-singular behavior throughout the manifold. Our rigorous mathematical analysis has yielded two novel classes of compact object solutions, designated as solutions 1 and 2, each exhibiting unique structural properties. The integration constants appearing in the metric potentials were fixed by matching our interior solutions to the exterior Reissner-Nordström spacetime through rigorous application of the Darmois junction conditions at the surface boundary r=R. Our comprehensive numerical analysis examined both solutions under certain values of *λ*, *γ* and *Q*. It is worthy mentioning here that our choice to focus the graphical analysis on LMC X-4 was motivated by its well-constrained observational parameters, allowing us to benchmark the models against a realistic compact object while exploring the effects of model parameters. On parameter variations, we have explicitly mapped out regimes where the solutions maintain physicality. For λ>0, the non-metricity contribution often leads to negative central densities or violates the weak energy condition, rendering solutions unphysical for compact objects consistent with prior f(Q) studies where positive *λ* amplifies repulsive effects similar to a cosmological constant, potentially destabilizing stellar equilibria. Conversely, for λ<−1, the models exhibit superluminal sound speeds near the core for stars with $M>2M_{⊙}$, indicating instability against perturbations. For *γ*, values exceeding 0.2 cause rapid pressure drops, violating the matching condition p(R)=0 smoothly, while γ<0 leads to inverted density profiles (increasing outward), which is unphysical. Radius variations (e.g., scaling R by ±20% for fixed *M*) show that overly compact configurations push the Buchdahl limit, but our models stay within bounds for λ≈−1. This analysis underscores that the solutions are not overly sensitive to the specific choice of LMC X-4 but generalize to a class of observed pulsars and X-ray binaries. The key findings are highlighted below.

- Our analysis confirms the metric components to remain well-defined, exhibit monotonic radial dependence, and satisfy core regularity conditions (Fig 1). Fig 2 verifies that all matter variables adhere to fundamental physical requirements for viable compact stars. The gradients shown in Fig 3 provide further validation of the solutions’ regularity.
- A self-gravitating system’s structure is fully characterized by its equation of state, linking pressure and energy density (Fig 4). Both mass function and compactness increase monotonically with radius, reaching their maximum at the surface, while the redshift vanishes in the center and grows outwards (Fig 5).
- Fig 6 reveals a definitive mass-moment of inertia correlation for the compact object solutions. Both configurations satisfy all energy conditions, confirming their physical viability as stellar interiors (Fig 7). The hydrostatic equilibrium analysis confirms the forces’ balance in both configurations (Fig 8).
- The sound speed remains subluminal and stable across all parameter variations. Both solutions demonstrate stability, with adiabatic index exceeding critical thresholds throughout their configurations. Further, both solution satisfy the Zeldovich criteria (Fig 9).
- Physically, the non-metricity scalar represents a deviation from the metric-compatible connection of GR, effectively rescaling the gravitational coupling in a way that mimics an additional geometric “stress” on the fluid distribution. In our linear model, the parameter *λ* modulates this effect: negative *λ* enhances attractive gravity in the stellar interior (increasing central densities by up to 30% compared to GR limits), while *γ* acts as a baseline shift similar to a vacuum energy term, influencing surface matching and compactness. The electric charge provides a repulsive counterbalance to gravity, reducing the required central pressure for equilibrium (by 10–15% in our models) and allowing for higher masses without collapse which is analogous to how charge stabilizes white dwarfs against electron degeneracy limits, but here applied to neutron-star-like densities.
- Observationally, our models predict mass-radius relations that deviate from GR by 5–10% for charged configurations, consistent with upper limits from pulsar timing), potentially testable with NICER or future ATHENA X-ray observations, which constrain radii to ~1km precision. The enhanced stability from negative *λ* could explain massive neutron stars without invoking exotic matter, aligning with GW170817 constraints on tidal deformability. Rather than claiming definitive superiority over GR, it must be stated that the models offer viable alternatives for interpreting compact object data, particularly in charged environments, and suggest targeted tests via electromagnetic counterparts to mergers or pulsar glitches.

Both solutions satisfy all critical stability criteria and physical requirements, confirming their viability as compact star models in modified gravity. Notably, the f(Q) gravity framework admits these compact solutions for specific parametric choices of the considered model.
